# Network pharmacology prediction and molecular docking-based strategy to explore the potential mechanism of Radix Astragali against hypopharyngeal carcinoma

**DOI:** 10.1038/s41598-023-50605-3

**Published:** 2024-01-04

**Authors:** Jianing Zhang, Lianhe Li

**Affiliations:** Department of Otorhinolaryngology-Head and Neck Surgery, Central Hospital of Chaoyang, Liaoning, 122000 China

**Keywords:** Drug screening, Target identification, Target validation, Head and neck cancer, Tumour biomarkers

## Abstract

To explore the anti-tumor effects of Radix Astragali on hypopharyngeal carcinoma and its mechanism. We have bioinformatically analyzed the potential targets of Radix Astragali and predicted the molecular mechanism of Radix Astragali treating of hypopharyngeal carcinoma. The binding process of the hub targets that could prolong the survival time of hypopharyngeal cancer patients with Radix Astragali was simulated by molecular docking. The results showed that 17 out of 36 hub targets could effectively improve the 5-year survival rate of hypopharyngeal cancer patients. Radix Astragali acts on hypopharyngeal carcinoma by regulating a signaling network formed by hub targets connecting multiple signaling pathways and is expected to become a drug for treating and prolonging hypopharyngeal carcinoma patients’ survival time.

## Introduction

Cancers of the hypopharynx are relatively rare, accounting for about 3% of all head and neck cancers^1^. Unfortunately, hypopharyngeal cancers have the worst prognosis of all head and neck cancers, with 5-year overall survival rates as low as 30–35%^2,3^. The cause of hypopharyngeal cancer is unclear and may be related to alcoholism, smoking, and viral infections (HPV, EBV)^4^. Due to its unique anatomical location, hypopharyngeal cancer has an insidious onset in the early stage. It often progresses to the middle or late stage when clinical symptoms appear, and its diagnosis relies on laryngoscopy, esophageal angiography, enhanced CT and MRI, etc. Its treatment mainly consists of surgery combined with radiotherapy and chemotherapy. However, due to the high degree of malignant and invasive hypopharyngeal cancer, the ease of lymphatic metastasis in the early stage, the early symptomatology is atypical, and the poor sensitivity of radiotherapy and chemotherapy, etc., which leads to unsatisfactory prognostic results and the 5-year survival rate of hypopharyngeal cancer is less than 30%^5^. As the pace of laryngeal preservation in advanced patients undergoing surgical treatment is low, and various complications such as pharyngeal fistula and ergotism will reduce the quality of survival and increase the burden of life of the patients, it is of great significance to search for new therapeutic options for hypopharyngeal cancer to improve the survival rate of patients with hypopharyngeal carcinoma.

Radix Astragali is one of the traditional Chinese medicines^6^. Traditional Chinese medicines are now widely used in clinical treatment^7^, both to act on a variety of targets^8,9^ to inhibit cancer and to alleviate the adverse effects of anticancer drugs^10^. Radix Astragali is widely used for its unique medicinal value and health benefits and is considered a traditional medicine and food^11^. In the toxicity study of Radix Astragali membranaceus extract, acute and subchronic oral toxicity tests were conducted in rats. In the critical toxicity study, a single dose of up to 5000 mg/kg was administered. In a 13-week subchronic toxicity study based on clinical signs, body weights, and necropsy findings, there were no deaths or toxic reactions^12^, which illustrates the safety of the Radix Astragali application. Whereas the interaction of Radix Astragali with other drugs may improve efficacy, astragaloside combined with apatinib mesylate significantly inhibited the growth of hepatocellular carcinoma. It promoted apoptosis^13^ and the combination with doxorubicin reduced myocardial toxicity^14^. It is extensively documented that the active ingredients of Radix Astragali affect various tumor biological behaviors, such as proliferation, apoptosis, migration, and infiltration^15–17^. As one of the bioactive substances of Radix Astragali, Radix Astragali polysaccharide (APS) significantly blocked the wnt/β-linker protein signaling pathway in a dose-dependent manner and thus inhibited the proliferation of breast cancer cells^18^. APS also inhibited epithelial-to-mesenchymal transition (EMT) and suppressed the expression of RAS/ERK and MAPK/NFkB pathway-related proteins in MSC-induced lung cancer cells^19^. APS is just one of the studies on biologically active substances related to anticancer in Radix Astragali. Astragaloside (AST) activates the pro-apoptotic pathway in colorectal cancer (CRC) cell lines, as evidenced by a decrease in the expression of the anti-apoptotic proteins Bcl-2, PARP and cysteinyl asparaginase 3 and an increase the expression of the pro-apoptotic proteins Bax, Bak, and Bad^20^. In the study of head and neck squamous cell carcinoma, it was found that Radix Astragali induced apoptosis in nasopharyngeal carcinoma cells, as evidenced by the elevated levels of cysteinyl asparaginase and Bax protein and the decreased level of Bcl-2 protein^21^, and APS significantly promoted the antiproliferative and apoptotic effects of cisplatin on nasopharyngeal carcinoma cells^22^. However, the literature has not reported its impact on hypopharyngeal carcinoma.

Based on systems biology, computer science, and bioinformatics, network pharmacology can efficiently and cost-effectively explore the relationship between drugs and diseases through high-throughput technologies, which enables the transition from the single-drug-single-target model of traditional pharmacology to the multi-drug-multi-target model^23–26^. Therefore, in this study, the effects of Radix Astragali on hypopharyngeal carcinoma and its specific mechanisms were systematically investigated using network pharmacology and molecular docking techniques.

## Methods

### Obtain the ingredients of Radix Astragali

The active ingredients of Radix Astragali were searched in the TCMSP database using the keyword “Radix Astragali”. The search criteria of OB > 30% and DL > 0.08 were used for the preliminary screening of the active ingredients of Radix Astragali. The obtained active ingredients were used as the effective active ingredients of Radix Astragali for subsequent data processing (OB and DL were selected according to the TCMSP website drug screening criteria recommendations and adjusted according to how many Radix Astragali targets were retrieved). TCMSP is a platform on the systemic pharmacology of herbal medicines, where we can get the relationship between drug, target and disease. The database provides information on identifying active ingredients, drug target networks, associated drug-target-disease networks, and pharmacokinetic properties such as oral bioavailability (OB)^27^ and drug-likeness (DL)^28^ for natural compounds. OB represents the percentage of an orally administered dose of an unchanged drug that reaches the systemic circulation, revealing the ADME process’s convergence. High oral bioavailability is often a key indicator to determine the drug-like properties of bioactive molecules as therapeutic agents. DL is a qualitative concept used in drug design to estimate how “drug-like” a prospective compound is, which helps optimize pharmacokinetic and pharmaceutical properties, such as solubility and chemical stability. The ‘drug-like’ level of the compounds is 0.18, which is used as a selection criterion for the ‘drug-like’ compounds in traditional Chinese herbs.

### Collecting Radix Astragali-related targets

Based on the active ingredients of Radix Astragali, Radix Astragali-related targets were screened in the TCMSP database, and the targets were searched in the ETCM database and Symmap database, respectively with “Radix Astragali” as the keyword and the targets obtained from the above three databases were integrated and de-duplicated. The targets obtained were regarded as Radix Astragali-related targets. ETCM is a comprehensive resource database of Chinese herbal medicines that went online in 2018 by the Chinese Academy of Traditional Chinese Medicine, bringing together information on a wide range of herbal medicines, herbal compounding, herbal chemical composition, drug targets, and related diseases. SymMap (Symptom Mapping) is a Chinese medicine evidence association database. The database herbs and the corresponding traditional Chinese medicine symptoms and correspond classic Chinese medicine symptoms to Western medicine symptoms, and includes diseases, herbal ingredients, drug targets associated with these symptoms, as well as the correlations between these six data types.

### Collection of hypopharyngeal carcinoma-related targets

We searched the Genecard database for hypopharyngeal carcinoma-related targets using the keyword “Hypopharyngeal Carcinoma”. The targets obtained were hypopharyngeal carcinoma-related targets. Genecards is a comprehensive, searchable database of genes where we can access information on almost all known human genes. Genecards automatically integrates resources from approximately 150 gene-centric databases, including genomics, transcriptomics, proteomics, genetics, clinical and functional information, and more.

### Construction of protein–protein interaction (PPI) networks

Venn diagram of Radix Astragali-related targets and hypopharyngeal carcinoma-related targets were plotted using R software (R 4.2.0), and the intersecting targets were used as potential targets of Radix Astragali for hypopharyngeal carcinoma. Then, we imported the potential targets of Radix Astragali for hypopharyngeal carcinoma into the String database, set the species as “Homo sapiens”, and set the confidence level as “0.9” to draw the PPI network.

### Screening of hub targets

We imported the PPI network into Cytoscape software (3.8.0) to further analyze the coefficients of the obtained marks, such as degree, Betweenness Centrality, and Closeness Centrality, and then, using degree ≥ degree median, Betweenness Centrality ≥ Closeness Centrality median and Closeness Centrality median as the screening criteria to screen the hub targets. The obtained targets were the hub targets.

### Plotting Kaplan–Meier (KM) curves

We imported the hub targets into the Kaplan–Meier Plotter online platform to plot KM curves. Since the platform does not have a separate database for Hypopharyngeal Carcinoma, we based the KM curves on the head and neck squamous cell carcinoma database, and P < 0.05 was significant. The division between high and low groups was chosen as the median for gene expression levels. The Kaplan–Meier Plotter database was constructed based on gene microarray and RNA-seq data from public databases such as GEO, EGA, and TCGA. It was used to integrate gene expression information and clinical prognostic values for meta-analysis and the study, discovery, and validation of survival-related molecular markers.

### Gene Ontology (GO) and Kyoto Encyclopedia of Genes and Genomes (KEGG) enrichment analysis

To analyze the potential functions and pathways of Radix Astragali acting on hypopharyngeal carcinoma, we used R software (R 4.2.0) to perform GO and KEGG enrichment analysis of potential targets of Radix Astragali for hypopharyngeal carcinoma, illustrating GO enrichment in terms of biological processes (BP), cellular components (CC), and molecular functions (MF). R-package-Bioconductor Cluster Profiler is an R package (R x64 4.0.3) widely used for gene bioinformatics analysis.

### Molecular docking

**Figure 1:**
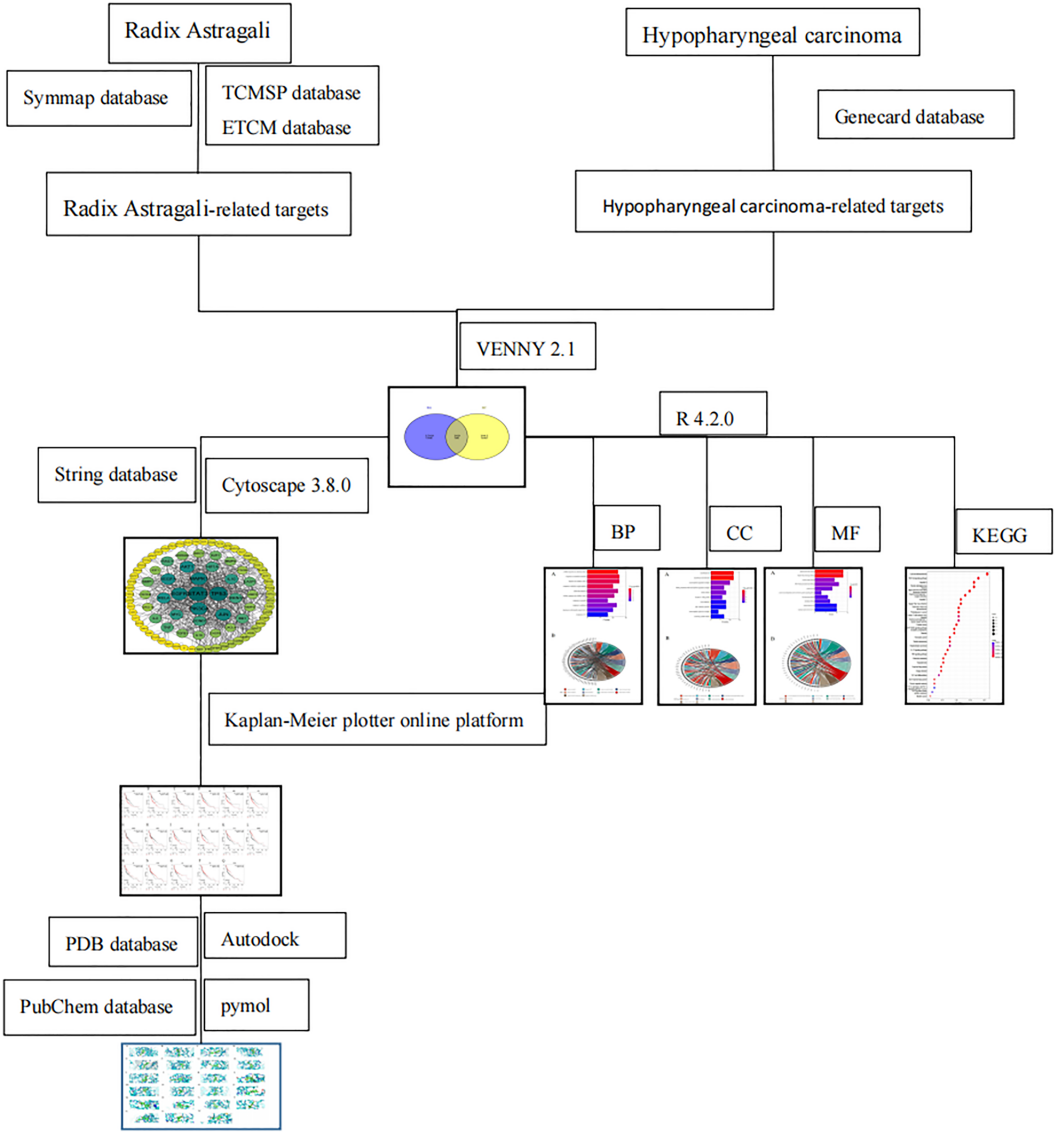
Network pharmacological study of Radix Astragali for treating hypopharyngeal carcinoma schematic diagram.

Autodock software is one of the software used for molecular docking. In this experiment, both Autodock 4 and Autodock Vina software were used for molecular docking, and Pymol software was used for molecular processing and visualization of docking results. The hub targets whose differential expression impacts the survival of head and neck squamous cell carcinoma patients and the corresponding active ingredients were used as molecular docking. 3D structures of the targets were obtained from the PDB database, and 3D structures of the active ingredients were obtained from the PubChm database. Autodock 4 and Pymol software were used to pre-process the target proteins, and Autodock Vina software was used for batch molecular docking. The technical roadmap of the entire study is shown in Fig. 1.

### Ethical approval

Because we use public and anonymous data, according to the ethics guidelines, neither informed consent nor approval of the ethics committee is required.

## Results

### Radix Astragali active ingredients

The active ingredients of Radix Astragali were searched in the TCMSP database using the keyword “Radix Astragali”, the active ingredients of Radix Astragali were screened using the screening criteria of OB > 30% and DL > 0.08. We finally obtained 20 active ingredients of Radix Astragali (Table 1).

**Table 1:** Characteristics of the active ingredients.

Mol ID	Molecule name	MW	OB (%)	DL
MOL000211	Mairin	456.78	55.38	0.78
MOL000239	Jaranol	314.31	50.83	0.29
MOL000296	Hederagenin	414.79	36.91	0.75
MOL000033	(24S)-24-Propylcholesta-5-ene-3beta-Ol	428.82	36.23	0.78
MOL000354	Isorhamnetin	316.28	49.6	0.31
MOL000371	3,9-Di-*O*-methylnissolin	314.36	53.74	0.48
MOL000374	5ʹ-Hydroxyiso-muronulatol-2ʹ,5ʹ-di-*O*-glucoside	642.67	41.72	0.69
MOL000378	7-*O*-Methylisomucronulatol	316.38	74.69	0.3
MOL000379	9,10-Dimethoxypterocarpan-3-*O*-β-d-glucoside	462.49	36.74	0.92
MOL000380	(6aR,11aR)-9,10-Dimethoxy-6a,11a-dihydro-6H-benzofurano[3,2-c]chromen-3-ol	300.33	64.26	0.42
MOL000387	Bifendate	418.38	31.1	0.67
MOL000392	Formononetin	268.28	69.67	0.21
MOL000398	Isoflavanone	316.33	109.99	0.3
MOL000417	Calycosin	284.28	47.75	0.24
MOL000422	Kaempferol	286.25	41.88	0.24
MOL000433	FA	441.45	68.96	0.71
MOL000438	(3R)-3-(2-Hydroxy-3,4-dimethoxyphenyl)chroman-7-ol	302.35	67.67	0.26
MOL000439	Isomucronulatol-7,2ʹ-di-*O*-glucosiole	626.67	49.28	0.62
MOL000442	1,7-Dihydroxy-3,9-dimethoxy pterocarpene	314.31	39.05	0.48
MOL000098	Quercetin	302.25	46.43	0.28

*ID* identity, *OB* oral bioavailability, *DL* drug likeness, *MW* molecule weight.

### Radix Astragali-related targets

We obtained 207 Radix Astragali-related targets from the TCMSP database, 271 Radix Astragali-related targets from the ETCM database, and 1484 Radix Astragali-related targets from the Symmap database. The above targets were integrated and de-duplicated to obtain 1589 Radix Astragali-related targets as the final Radix Astragali-related targets for further processing.

### Hypopharyngeal carcinoma-related targets

We used the keyword “Hypopharyngeal Carcinoma” in the Genecard database of hypopharyngeal carcinoma-related targets. We obtained 1050 hypopharyngeal carcinoma-related targets, and the targets obtained above were regarded as hypopharyngeal carcinoma-related targets.

### PPI network

**Figure 2:**
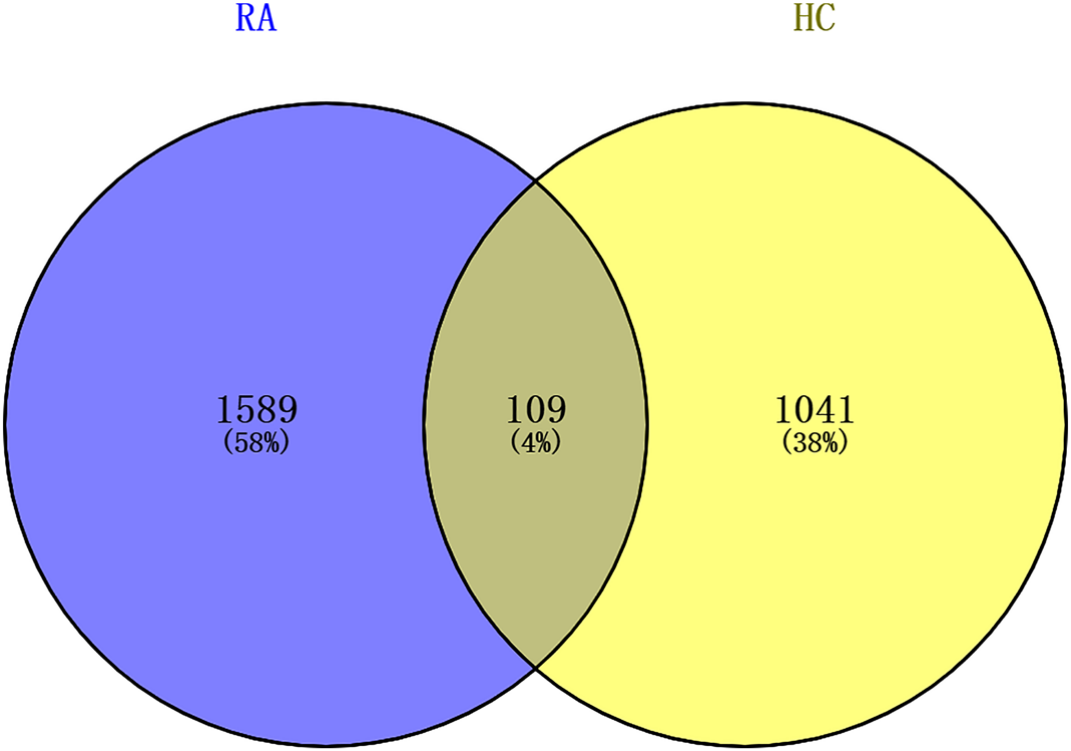
The Venn diagram about the target of Radix Astragali and the target of hypopharyngeal carcinoma. The blue circle represents the target of Radix Astragali, and the yellow circle represents the target of hypopharyngeal carcinoma. The intersection of the two circles represents the target of Radix Astragali for hypopharyngeal carcinoma.

**Figure 3:**
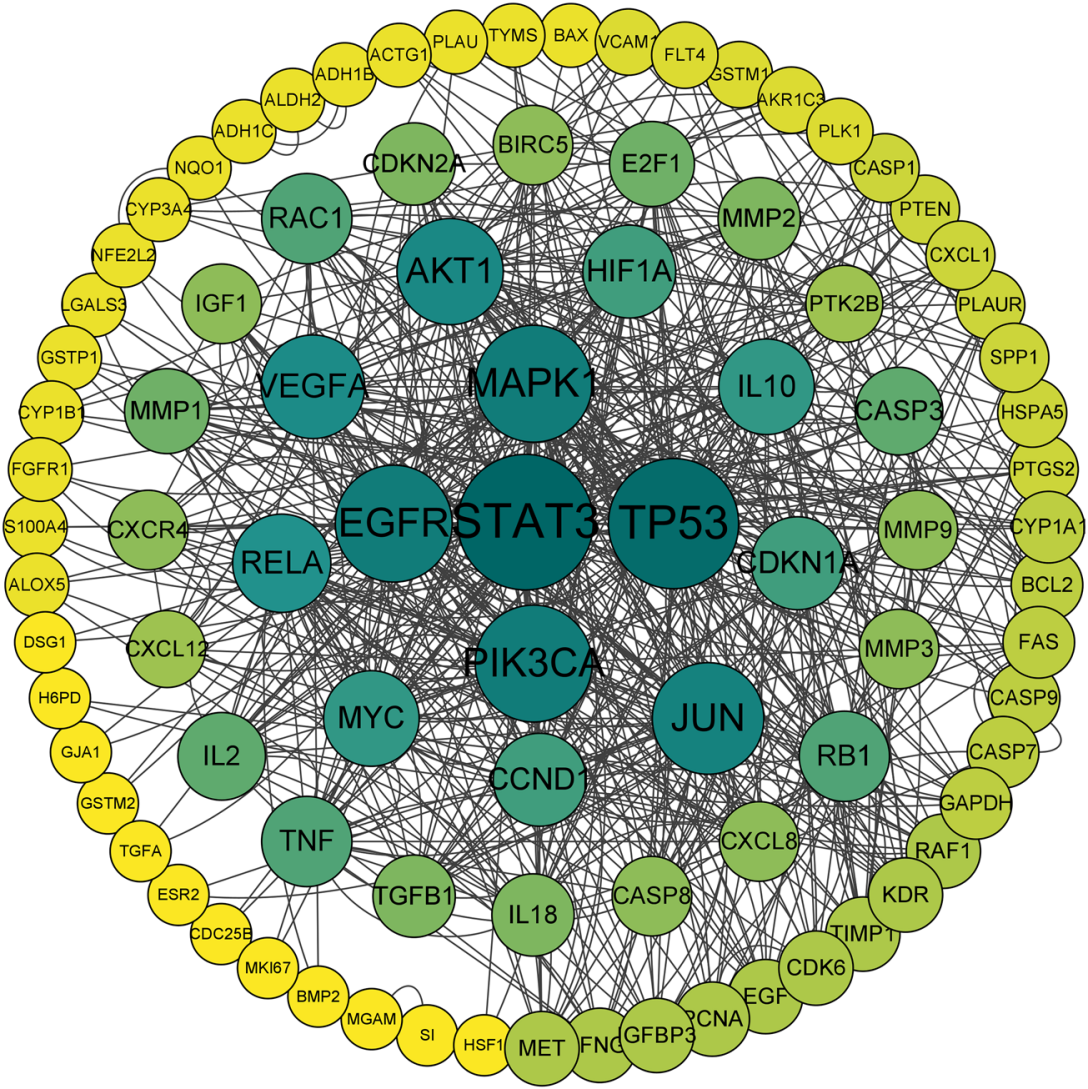
PPI network of Radix Astragali in the treatment of hypopharyngeal carcinoma. The nodes represent potential therapeutic targets of Radix Astragali against hypopharyngeal carcinoma. The larger the node, the higher the corresponding target degree and the more connections to other nodes.

We made a Venn diagram with Radix Astragali-related and hypopharyngeal carcinoma-related targets (Fig. 2). The intersection of the two was the target related to Radix Astragali for hypopharyngeal cancer, and a total of 109 potential targets of Radix Astragali for hypopharyngeal cancer were obtained. The potential targets of Radix Astragali for hypopharyngeal carcinoma were imported into the String database to construct a PPI network and imported into Cytoscape software for processing and analysis (Fig. 3), with Degree ≥ 10, Betweenness Centrality ≥ 0.00399091 and Closeness Centrality ≥ 0.391509434 as the screening criteria for screening hub targets to obtain hub targets, and a total of 20 hub targets were obtained (Table 2).

**Table 2:** Characteristics of the hub gene.

Name	Betweenness centrality	Closeness centrality	Degree
TP53	0.190369985	0.522012579	54
JUN	0.173872843	0.506097561	40
STAT3	0.146888138	0.535483871	58
EGFR	0.143920539	0.49112426	44
MAPK1	0.12219857	0.512345679	44
PIK3CA	0.075504206	0.485380117	44
AKT1	0.062814915	0.488235294	36
VEGFA	0.061834647	0.456043956	34
RAC1	0.049798266	0.458563536	24
CASP3	0.047023398	0.410891089	22
BIRC5	0.034272603	0.412935323	16
GAPDH	0.033755795	0.397129187	10
TNF	0.022590881	0.419191919	24
CDKN1A	0.022019016	0.451086957	26
RB1	0.021168384	0.439153439	24
CCND1	0.020340795	0.443850267	26
PTK2B	0.019186977	0.395238095	14
RELA	0.018247514	0.471590909	30
IL10	0.017616548	0.402912621	28
MYC	0.015986422	0.468926554	28
HIF1A	0.014843737	0.474285714	26
MMP3	0.014480116	0.393364929	16
IL2	0.013604601	0.448648649	22
CASP8	0.011876765	0.410891089	16
CXCR4	0.011067351	0.417085427	16
IGFBP3	0.008088196	0.406862745	12
TGFB1	0.008030993	0.415	18
CXCL12	0.007755732	0.406862745	14
IGF1	0.006442284	0.400966184	16
FAS	0.005908254	0.406862745	10
MET	0.005241729	0.415	12
CDKN2A	0.005091	0.404878049	18
BCL2	0.004599699	0.408866995	10
MMP1	0.004590138	0.395238095	20

### Plotting Kaplan–Meier (KM) curves

**Figure 4:**
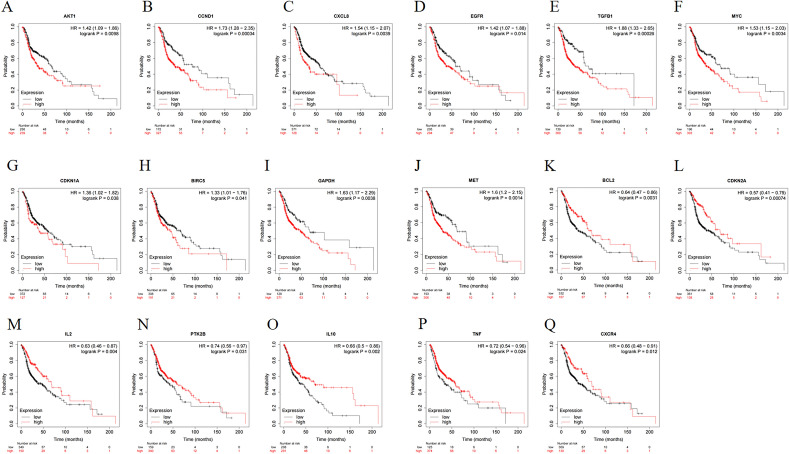
The Kaplan–Meier curves. Prolonged median survival in patients with hypopharyngeal cancer when (**A**) (AKT1), (**B**) (CCND1), (**C**) (CXCL8), (**D**) (EGFR), (**E**) (TGFB1), (**F**) (MYC), (**G**) (CDKN1A), (**H**) (BIRC5), (**I**) (GAPDH), and (**J**) (MET) is overexpressed or (**K**) (BCL2), (**L**) (CDKN2A), (**M**) (IL2), (**O**) (IL10), (**P**) (TNF), and (**Q**) (CXCR4) expression is decreased.

The hub targets were imported separately into The Kaplan–Meier Plotter online platform, and KM curves were plotted. The results showed that differential expression of a total of 17 hub genes had impacted the overall survival of patients with head and neck squamous cell carcinoma (Fig. 4). Targets that were genetically upregulated to improve overall survival in patients with head and neck squamous cell carcinoma included Interleukin-2 (IL2), Interleukin-1 (IL10), Apoptosis regulator Bcl-2 (BCL2), Tumor suppressor ARF (CDKN2A), C-X-C chemokine receptor type 4 (CXCR4), non-specific protein-tyrosine kinase (PTK2B), and Tumor necrosis factor (TNF); targets that were able to improve patient overall survival when gene expression was downregulated were non-specific protein-tyrosine kinase (AKT1), G1/S-specific cyclin-D1 (CCND1), C-X-C chemokine receptor type 8 (CXCL8), Epidermal growth factor receptor (EGFR), Cip1-interacting zinc finger protein (CDKN1A), Baculoviral IAP repeat-containing protein 5 (BIRC5), Glyceraldehyde-3-phosphate dehydrogenase (GAPDH), Hepatocyte growth factor receptor (MET), Myc proto-oncogene protein (MYC), and Transforming growth factor beta-1 proprotein (TGFB1).

### GO and KEGG enrichment analysis

**Figure 5:**
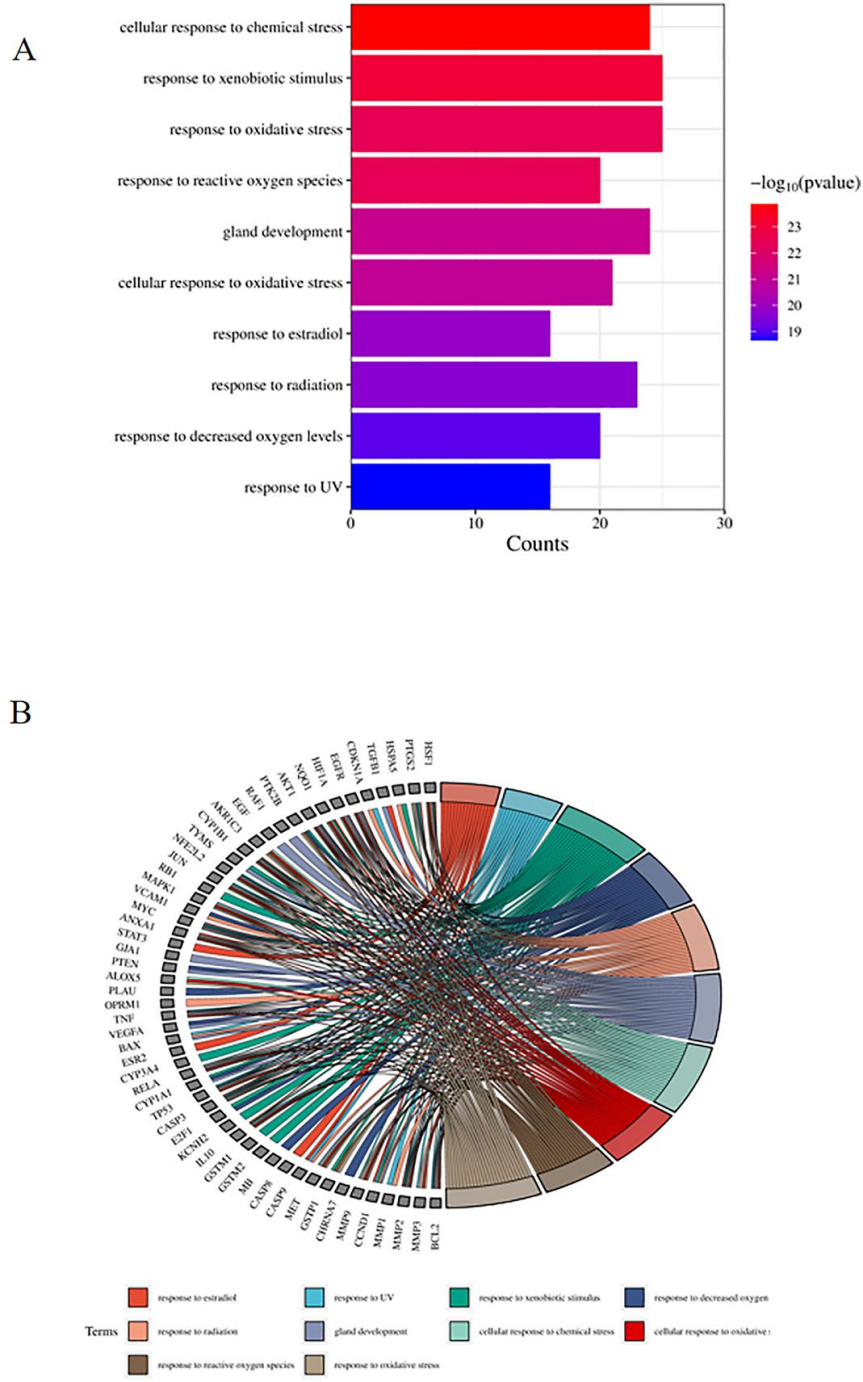
Top ten significant biological process (BP) entries. (**A**) GO enrichment analysis of therapeutic targets for biological process. (**B**) Relationship between the therapeutic targets and biological process.

**Figure 6:**
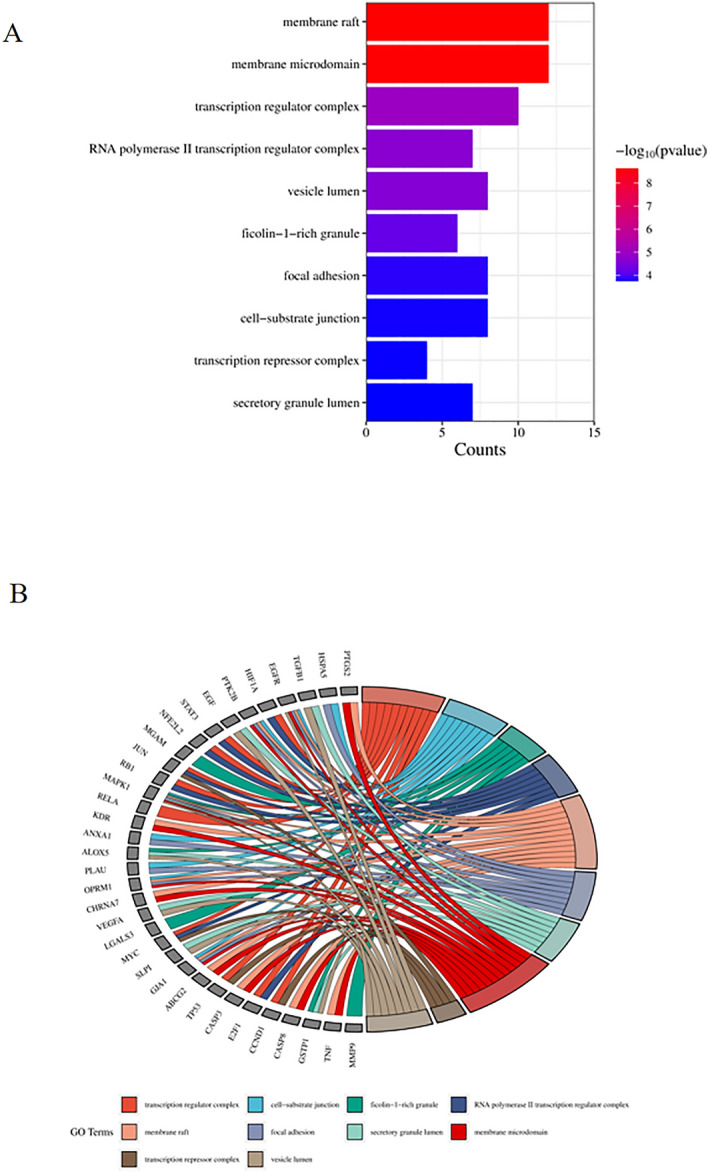
Top ten significant cell component (CC) entries. (**A**) GO enrichment analysis of therapeutic targets for cell components. (**B**) Relationship between the therapeutic targets and cell component.

**Figure 7:**
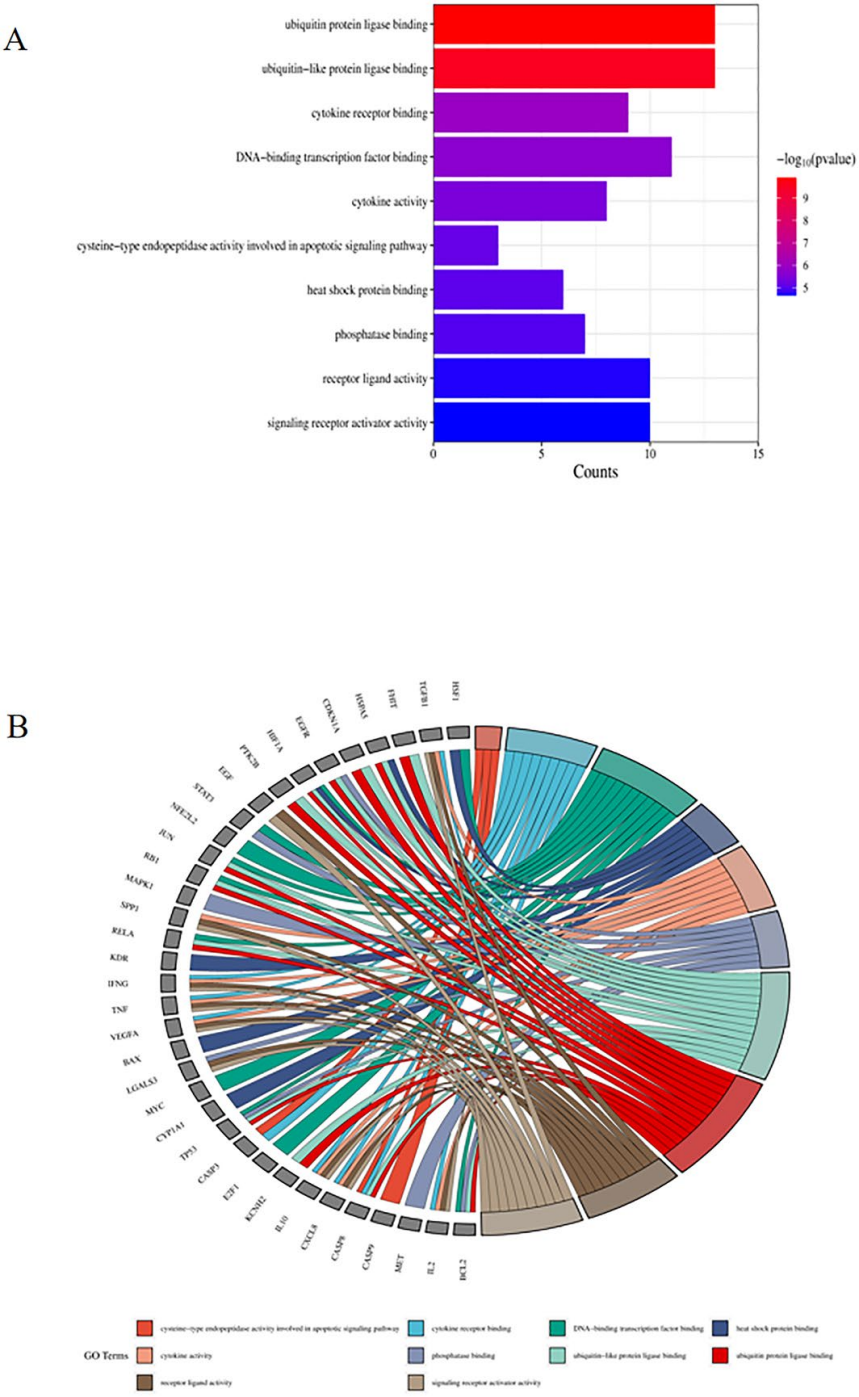
Top ten significant molecular function (MF) entries. (**A**) GO enrichment analysis of therapeutic targets for molecular function. (**B**) Relationship between the therapeutic targets and molecular function.

**Figure 8:**
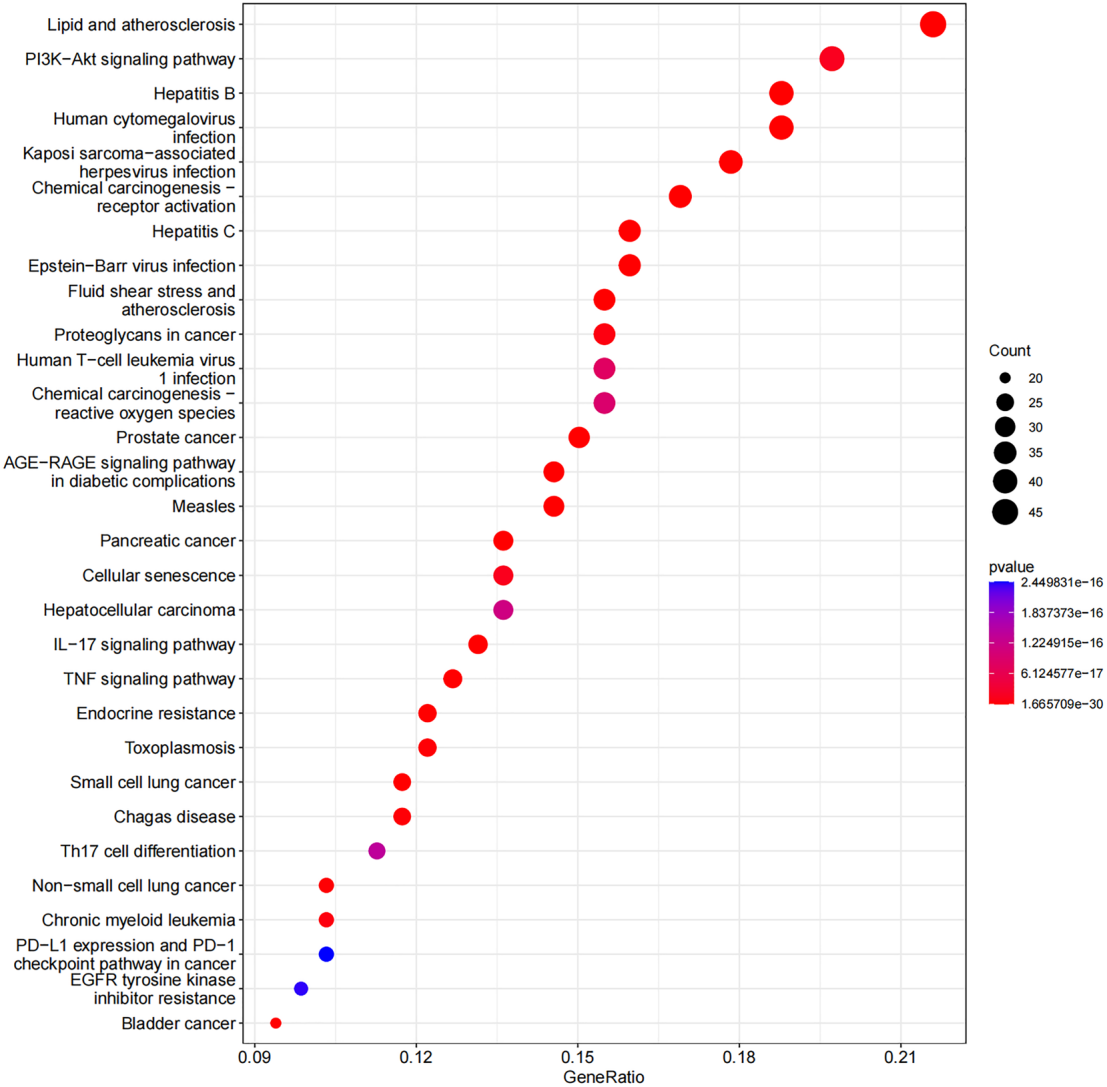
KEGG enrichment analysis for therapeutic targets.

To investigate the potential function of Radix Astragali for hypopharyngeal carcinoma, we subjected the potential targets of Radix Astragali for hypopharyngeal carcinoma to GO enrichment analysis, and we show the results of GO enrichment analysis in terms of BP, CC, and MF. P-values are arranged from most minor to most significant, and the top 10 BP, CC, and MF are shown in Figs. 5A,B, 6A,B and 7A,B. Figures 5B, 6B and 7B highlight the genes and relationship between functions. To explore the potential pathways of Radix Astragali for hypopharyngeal carcinoma, we subjected the potential targets of Radix Astragali for hypopharyngeal carcinoma to KEGG enrichment analysis, with the P-values arranged from smallest to largest, and the top 30 results of the enrichment results are displayed in Fig. 8.

### Molecular docking

**Figure 9:**
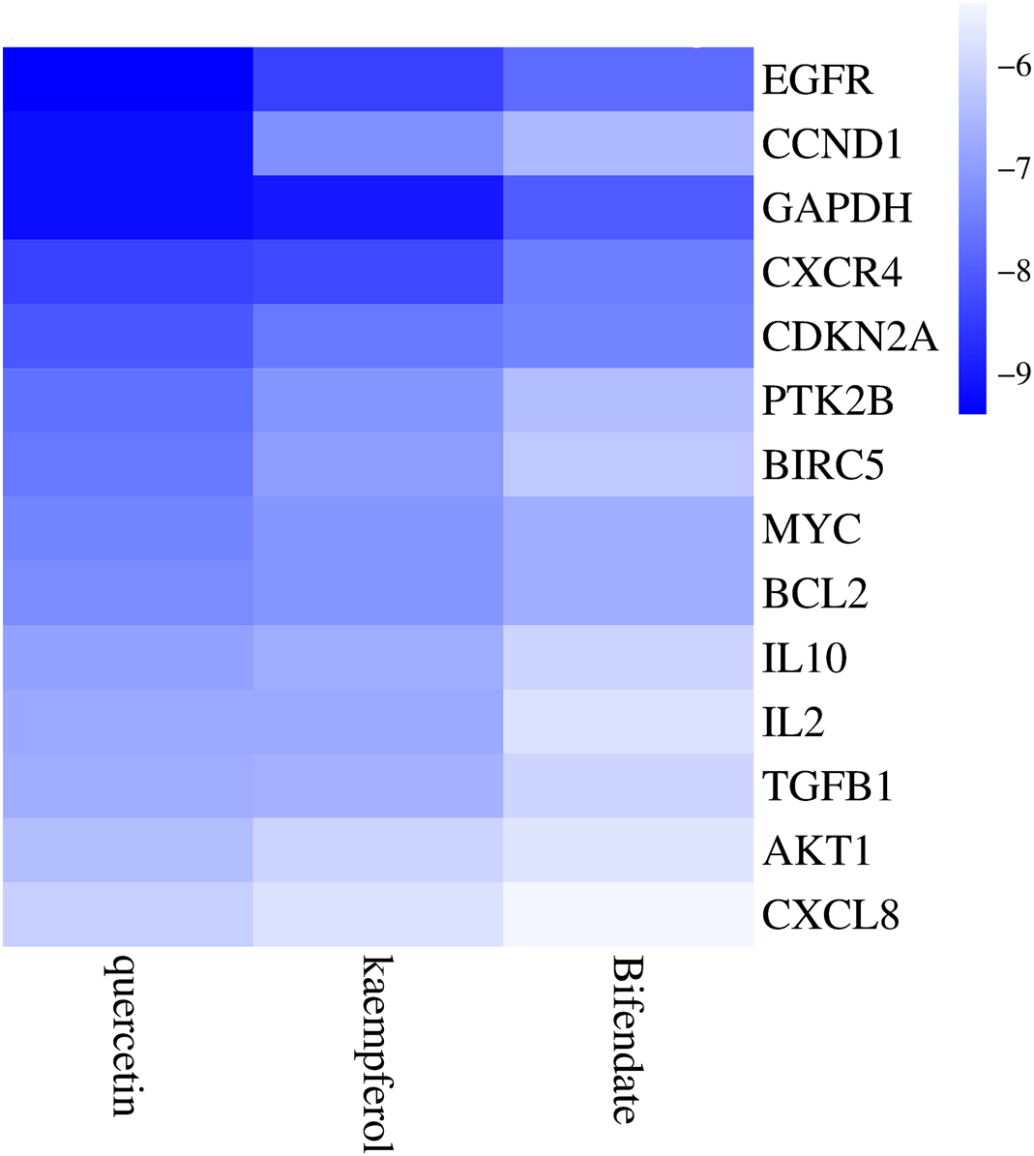
Heatmaps of the docking scores of hub targets combined with the corresponding bioactive compound of Radix Astragali. The darker the blue, the more free energy the bioactive ingredient has to bind to the hub targets.

**Figure 10:**
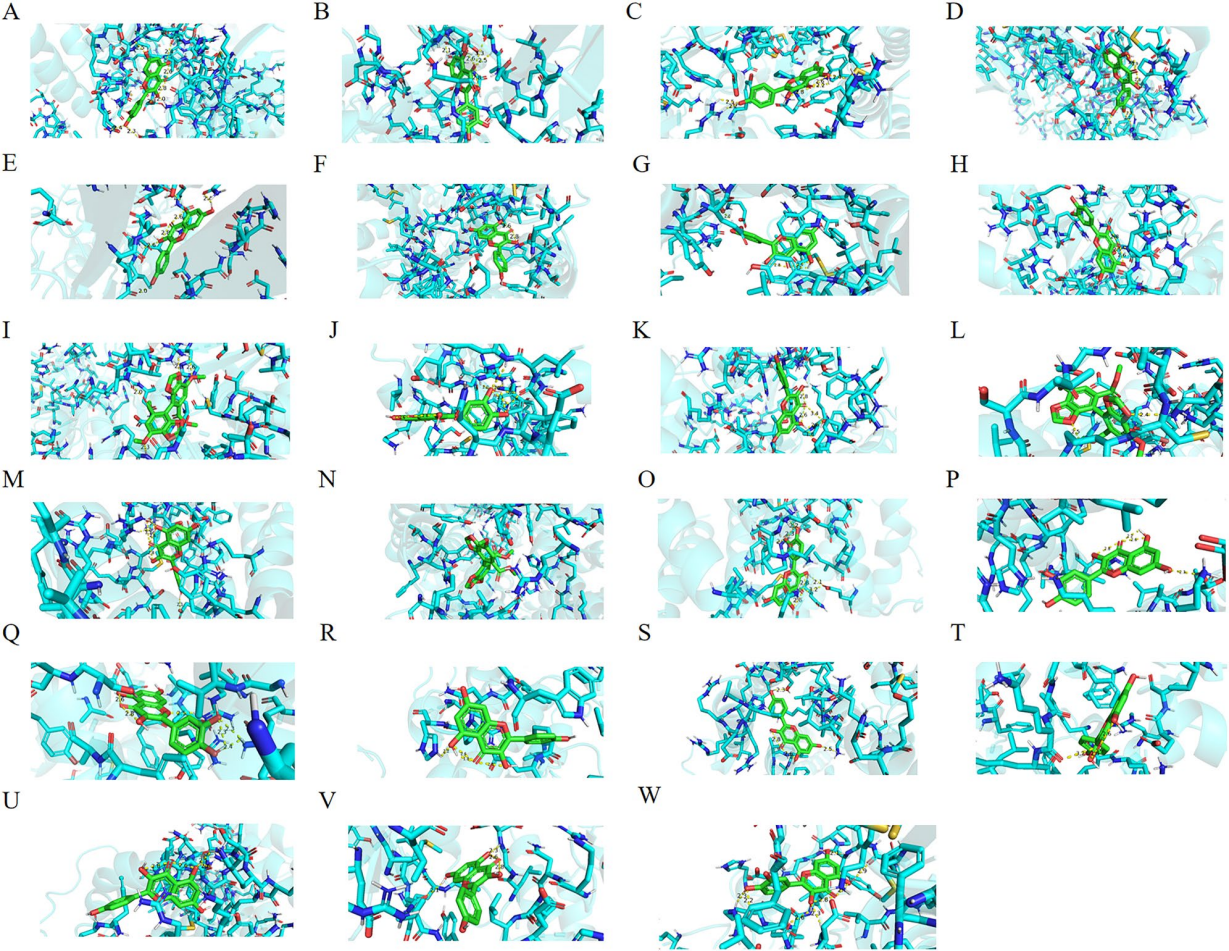
The significant molecular docking. (**A**) (CCND1, quercetin, − 9.2 kcal/mol); (**B**) (GAPDH, quercetin, − 9.2 kcal/mol); (**C**) (EGFR, kaempferol, − 9 kcal/mol); (**D**) (EGFR, quercetin, − 8.8 kcal/mol); (**E**) (GAPDH, kaempferol, − 8.8 kcal/mol); (**F**) (CXCR4, kaempferol, − 8.3 kcal/mol), (**G**) (CXCR4, quercetin, − 8.1 kcal/mol); (**H**) (CDKN2A, quercetin, − 8 kcal/mol), (**I**) (GAPDH, bifendate, − 7.9 kcal/mol); (**J**) (BIRC5, quercetin, − 7.6 kcal/mol), (**K**) (CDKN2A, kaempferol, − 7.6 kcal/mol); (**L**) (EGFR, bifendate, − 7.4 kcal/mol), (**M**) (BCL2, kaempferol, − 7.3 kcal/mol); (**N**) (CXCR4, bifendate, − 7.3 kcal/mol); (**O**) (IL2, quercetin, − 7.3 kcal/mol); (**P**) (MYC, quercetin, − 7.3 kcal/mol); (**Q**) (BCL2, quercetin, − 7.2 kcal/mol); (**R**) (CCND1, kaempferol, − 7.2 kcal/mol); (**S**) (MYC, kaempferol, − 7.2 kcal/mol); (**T**) (BIRC5, kaempferol, − 7.1 kcal/mol); (**U**) (IL2, kaempferol, − 7.1 kcal/mol); (**V**) (PTK2B, kaempferol, − 7.1 kcal/mol); (**W**) (PTK2B, quercetin, − 7.1 kcal/mol).

To simulate the process of mutual binding between the hub target whose differential expression of the target has an impact on the survival rate of head and neck squamous cell carcinoma patients, and the corresponding active ingredient of Radix Astragali, we made a molecular docking, and the docking with a release free energy <  − 7 kcal/mol indicated that the complementary active ingredient and the target and bind effectively in the natural state. Based on the docking results, we plotted the heat map (Fig. 9), and we visualized the docking results for the docking with a release free energy <  − 7 kcal/mol (Fig. 10) and the basic information of the docking results is displayed in Table 3.

**Table 3:** Information on the docking results of the significant molecules.

Receptor	Ligands	Free energy (kcal/mol)	Corresponding serial numbers in Fig. 10
CCND1	Quercetin	− 9.2	A
GAPDH	Quercetin	− 9.2	B
EGFR	Kaempferol	− 9	C
EGFR	Quercetin	− 8.8	D
GAPDH	Kaempferol	− 8.8	E
CXCR4	Kaempferol	− 8.3	F
CXCR4	Quercetin	− 8.1	G
CDKN2A	Quercetin	− 8	H
GAPDH	Bifendate	− 7.9	I
BIRC5	Quercetin	− 7.6	J
CDKN2A	Kaempferol	− 7.6	K
EGFR	Bifendate	− 7.4	L
BCL2	Kaempferol	− 7.3	M
CXCR4	Bifendate	− 7.3	N
IL2	Quercetin	− 7.3	O
MYC	Quercetin	− 7.3	P
BCL2	Quercetin	− 7.2	Q
CCND1	Kaempferol	− 7.2	R
MYC	Kaempferol	− 7.2	S
BIRC5	Kaempferol	− 7.1	T
IL2	Kaempferol	− 7.1	U
PTK2B	Kaempferol	− 7.1	V
PTK2B	Quercetin	− 7.1	W

## Discussion

Hypopharyngeal carcinoma is a head and neck tumor, accounting for about 3% of head and neck tumors^29^. Hypopharyngeal carcinoma is highly malignant and has a poor healing process. Patients are often found to have reached an advanced stage and distant metastasis, with an overall survival rate of 30–35%^1,2^. Although people are looking for new treatments to improve the outcome of hypopharyngeal carcinoma, the outcome of hypopharyngeal carcinoma has yet to be greatly improved in recent years^3,30^, and new treatment strategies are urgently needed to improve the overall survival rate of hypopharyngeal carcinoma patients. Radix Astragali is one of the traditional Chinese medicines, and its various active ingredients have been proven to affect the biological behavior of tumors, such as proliferation, apoptosis, migration, and infiltration. Still, its effect on hypopharyngeal cancer and its mechanism has not been reported in the literature^15–17^. For these reasons, this study investigated the effect of Radix Astragali on hypopharyngeal carcinoma and the mechanism using network pharmacology and molecular docking techniques. In this study, we obtained Radix Astragalis and hypopharyngeal carcinoma-related targets from several databases. We intersected the above two targets, and the intersected targets were used as potential therapeutic targets of Radix Astragali for hypopharyngeal carcinoma for constructing a PPI network, GO, and KEGG enrichment analysis. The pivotal targets were obtained by PPI network analysis, and the KM curves of the pivotal targets were constructed to screen 17 pivotal targets that could influence the overall survival rate of hypopharyngeal carcinoma. Among them, the hub targets whose up-regulated expression could improve the overall survival rate of hypopharyngeal cancer patients included IL2, IL10, BCL2, CDKN2A, CXCR4, PTK2B, and TNF; the hub targets whose down-regulated expression could improve the overall survival rate of hypopharyngeal cancer patients included AKT1, CCND1, CXCL8, EGFR, CDKN1A, BIRC5, GAPDH, MET, MYC, and TGFB1. KEGG results showed that Radix Astragali acted on hypopharyngeal carcinoma through multiple signaling pathways, such as PI3K-Akt signaling pathway, IL-17 signaling pathway, TNF signaling pathway, and PD-L1 expression and PD-1 checkpoint pathway in cancer. These signaling pathways are well documented to affect the biological behavior of various tumors, such as the PI3K-Akt signaling pathway, which has been documented to promote apoptosis, inhibit tumor cell proliferation, and migrate a variety of cells. These signaling pathways have been extensively studied to influence the biological behavior of various tumors. For example, the PI3K-Akt signaling pathway has been shown in the literature to promote apoptosis, inhibit tumor cell proliferation, and migrate in a variety of cells^31–34^, The IL-17 signaling pathway mediates tumor growth^35,36^, and the TNF signaling pathway inhibits tumor metastasis when activated^37,38^, etc. Meanwhile, the KEGG results showed that the hub targets are involved in signaling multiple signaling pathways, such as AKT1 in the PI3K-Akt signaling pathway, TNF signaling pathway, PD-L1 expression, and PD-1 checkpoint pathway in cancer. For example, AKT1 is involved in the PI3K-Akt signaling pathway, TNF signaling pathway, PD-L1 expression, and PD-1 checkpoint pathway in cancer, TNF is involved in the L-17 signaling pathway, TNF signaling pathway, etc. By signaling multiple signaling pathways, the hub target connects multiple signaling pathways into an interoperable signaling network. Molecular docking results showed that quercetin and other active ingredients could freely bind to the hub targets, affecting the overall survival of hypopharyngeal carcinoma patients, with quercetin effectively docking with the most hub targets and Radix Astragali acting as the most promising target for hypopharyngeal carcinoma to improve the overall survival of patients. Although few studies have been conducted on the clinical use of Radix Astragali in the treatment of hypopharyngeal cancer, they have shown that Radix Astragali is one of the most effective herbs for reducing mortality in patients with advanced breast cancer, that Radix Astragali compounds are more effective in the treatment of hepatitis than controls; and that the combination of Radix Astragali and glucocorticoids (GCs) not only reduces the dosage of GCs but also mitigates the side effects caused by GCs. Quercetin is the main representative of the flavonol flavonoid subclass, and its anticancer effects have been demonstrated in various cancers. Quercetin inhibited the proliferation of colon, ovarian, and breast cancer cell lines^39,40^. Quercetin can also inhibit breast cancer progression through Akt-mTOR pathway-mediated autophagy-induced inhibition of cell migration and glycolysis^41^. Similar mechanisms have been found in quercetin-activated autophagy against gastric carcinogenesis^42^. Quercetin also has anticancer potential in head and neck squamous cell carcinoma. Studies have shown that quercetin inhibits the migration and invasion of head and neck squamous cell carcinoma cells by suppressing the expression of MMP-2 and MMP-9^43^. Quercetin increases the expression levels of cytochrome c, apoptosis-inducing factor, and nucleic acid endonuclease G, thereby promoting apoptosis in oral cancer cells^44^. Quercetin also inhibits the growth of oral squamous cell carcinoma xenograft tumors by inducing miR-22 expression and inhibiting the WNT1/β-linker pathway in vivo^45^. Quercetin induced apoptosis in laryngeal cancer cells by inhibiting Akt/PKB phosphorylation and significantly decreasing anti-apoptotic Bcl-2 and Bcl-XL, thus acting as a chemosensitizer to cisplatin. Compared with cisplatin alone, quercetin combined with cisplatin increased the apoptosis rate from 18.7 to 42.2% in the laryngeal cancer HEP-2 cell line^46^. Therefore, combining previous studies with our results, quercetin may inhibit the migration and invasion of hypopharyngeal squamous cell carcinoma cells by regulating the PI3K-Akt signaling pathway, thus prolonging the overall survival of patients with hypopharyngeal carcinoma, which is also one of the directions of our subsequent studies.

We discovered Radix Astragali’s regulatory effect on hypopharyngeal carcinoma through pharmacological and molecular docking techniques. We are the first to find the above results. As we all know, network pharmacology is an efficient tool for the investigation of drug mechanisms. However, it is undeniable that the present study still has some limitations: firstly, In vitro and in vivo experiments were not conducted in this study, and the effect of Radix Astragali on hypopharyngeal carcinoma in the in vitro and in vivo situations and how quercetin binds to and modulates central targets were not explored; which we will continue to study in the next step; secondly, each database has a different focus, and there are differences between databases; If conditions permit, we will further employ Molecular quantification techniques (such as mass spectrometry) to confirm Quercetin’s presence to ensure the accuracy of the experimental results; therefore, there may be potential risks in the joint analysis of multiple databases. Thus, the discovery of new targets and pathways still needs to be done through basic laboratory experiments.

## Conclusion

Through network pharmacology and molecular docking techniques, we found that the active ingredients of Radix Astragali act on multiple hub targets to regulate various signaling pathways connected to the hub targets to form a signaling network, through which Radix Astragali modulates the biological behavior of hypopharyngeal carcinoma and improves the overall survival rate of hypopharyngeal carcinoma patients. This process may be responsible for the inhibition of migration and invasion of hypopharyngeal squamous cell carcinoma cells by quercetin through the regulation of the PI3K-Akt signaling pathway. Among the active ingredients of Radix Astragali, quercetin has the most potential to become a therapeutic agent for hypopharyngeal carcinoma and to improve the overall survival rate of hypopharyngeal carcinoma patients.

## Data Availability

The original contributions presented in the study are included in the article Material. Further inquiries can be directed to the corresponding authors.

## Abbreviations

BP: Biological processes
CC: Cellular components
DL: Drug likeness
GO: Gene Ontology
KEGG: Kyoto Encyclopedia of Genes and Genomes
KM: Kaplan–Meier
MF: Molecular functions
OB: Oral bioavailability
PPI: Protein–protein interaction
TNF: Tumor necrosis factor
MYC: Myc proto-oncogene protein
IL2: Interleukin-2
IL10: Interleukin-10
BCL2: Apoptosis regulator Bcl-2
CDKN2A: Tumor suppressor ARF
CXCR4: C-X-C chemokine receptor type 4
PTK2B: Non-specific protein-tyrosine kinase
AKT1: Non-specific protein-tyrosine kinase
CCND1: G1/S-specific cyclin-D1
CXCL8: C-X-C chemokine receptor type 8
EGFR: Epidermal Growth Factor Receptor
CDKN1A: Cip1-interacting zinc finger protein
BIRC5: Baculoviral IAP repeat-containing protein 5
GAPDH: Glyceraldehyde-3-phosphate dehydrogenase
MET: Hepatocyte growth factor receptor
TGFB1: Transforming growth factor beta-1 proprotein
